# Mesenchymal stem cells-derived exosomes as a promising new approach for the treatment of infertility caused by polycystic ovary syndrome

**DOI:** 10.3389/fphar.2022.1021581

**Published:** 2022-10-10

**Authors:** Mahin Izadi, Mohammad Ebrahim Rezvani, Ali Aliabadi, Mahdieh Karimi, Behrouz Aflatoonian

**Affiliations:** ^1^ Research and Clinical Center for Infertility, Yazd Reproductive Sciences Institute, Shahid Sadoughi University of Medical Sciences, Yazd, Iran; ^2^ Department of Reproductive Biology, School of Medicine, Shahid Sadoughi University of Medical Sciences, Yazd, Iran; ^3^ Department of Physiology, School of Medicine, Shahid Sadoughi University of Medical Sciences, Yazd, Iran; ^4^ Stem Cell Biology Research Center, Yazd Reproductive Sciences Institute, Shahid Sadoughi University of Medical Sciences, Yazd, Iran

**Keywords:** mesenchymal stem cell, exosome, extracellular vesicle, apoptosis, chronic inflammation

## Abstract

Polycystic ovary syndrome (PCOS) is a multifactorial metabolic and most common endocrine disorder that its prevalence, depending on different methods of evaluating PCOS traits, varies from 4% to 21%. Chronic low-grade inflammation and irregular apoptosis of granulosa cells play a crucial role in the pathogenesis of PCOS infertility. Mesenchymal stem cells (MSCs)-derived exosomes and extracellular vesicles (EVs) are lipid bilayer complexes that act as a means of intercellular transferring of proteins, lipids, DNA and different types of RNAs. It seems that this nanoparticles have therapeutic effects on the PCOS ovary such as regulating immunity response, anti-inflammatory (local and systemic) and suppress of granulosa cells (GCs) apoptosis. Although there are few studies demonstrating the effects of exosomes on PCOS and their exact mechanisms is still unknown, in the present study we reviewed the available studies of the functions of MSC-derived exosome, EVs and secretome on apoptosis of granulosa cells and inflammation in the ovary. Therefore, the novel cell-free therapeutic approaches for PCOS were suggested in this study.

## Introduction

Polycystic ovary syndrome (PCOS) is a multifactorial metabolic disease and common endocrine condition that leads to increased production of androgens, decreased production of the estrogens and progesterone and consequences of infertility (Xu and Qiao, 2022). Common biochemical hallmarks of polycystic ovary syndrome are the absence of ovulation with high levels of androgens, luteinizing hormone (LH), luteinizing hormone/follicle-stimulating hormone ratios while follicle-stimulating hormone (FSH) remains normal or low (Eid et al., 2005). The global prevalence of this disorder varies from 4% to 21% depending on different methods of evaluating PCOS traits and diagnostic criteria (Lizneva et al., 2016), which can be recognized as the most common cause of infertility or failed birth in recent years (Rotterdam, 2004; Lizneva et al., 2016). It seems the principal ovarian consequences of PCOS are growth arrest in the early antral follicles and abnormal folliculogenesis (Franks et al., 2008). Although the current treatments include various gonadotropin (Artini et al., 1996), clomiphene citrate (Legro et al., 2007) and metformin (Sam and Dunaif, 2003), but, it has been pointed out that each of these treatments have various advantages and disadvantages (Elnashar et al., 2006; Legro et al., 2007). Therefore, alternative and non-invasive treatments improving follicle growth, resumption of oocyte maturation and different leading factors of PCOS are needed. There is evidence that mesenchymal stem cells (MSCs) have anti-inflammatory, fibrogenesis inhibiting, antioxidant, and regenerative effects (Zhao et al., 2019). These roles can give to the MSCs a potential therapeutic application in various abnormalities such as the female reproductive disorders (Mutlu et al., 2015; He et al., 2018).

In addition to intercellular interactions such as autocrine, paracrine or endocrine signaling, recently, extracellular vesicles (EVs) as a new tool for intercellular communication has attracted the attention of researchers. Although, some researchers consider the secretion of EVs as a mechanism of the cell to dispose of useless molecules (Van der Pol et al., 2012). But using the extracellular vesicles, various active biomolecules including nucleic acids, proteins and lipids can be transferred from origin cells to target cells (György et al., 2011; Koniusz et al., 2016). Precise characterization of the EVs content has opened up their promising applications in diagnosis and therapy, as well as the development of innovative drug delivery systems (Barile and Vassalli, 2017). According to their biosynthesis mechanism and size, those can be divided in to microvesicles (50–3000 nm), exosomes (40–100 nm) and apoptotic bodies (800–5000 nm) (Yamamoto et al., 2016). Origin-based contents, genetic materials and ability to content shuttling to other cells make exosomes as an attractive research subject for manipulating the functions of different cells locally and/or remotely (Han et al., 2016). In various physiological and pathological processes including reproduction, gametogenesis, embryogenesis and differentiation, exosomes are secreted by most cell types into the extracellular environment and have been detected in various body fluids (Raposo and Stoorvogel, 2013; Machtinger et al., 2016) so that they act as a means of transferring proteins, lipids, DNA and diversity of RNA species between cells (Barile and Vassalli, 2017). The presence of EVs in reproductive bio-fluids such as follicular fluid and ovarian fluid shows their role in the intercellular communication necessary for the proper functioning of the reproductive system (Machtinger et al., 2016). Considering the positive role of MSC-derived exosomes, the goal of this study is to review the available reports on their role in treatments of the various reproductive processes and present a potential role of the exosome in *in vitro* maturation of oocyte and the improve of infertility in PCOS women.

### Mesenchymal stem cells-derived exosomes

Growing evidence from a various experimental and clinical trials support the effectiveness of MSCs on treating different diseases such as renal fibrosis, cardiovascular disorders, neurological diseases and female reproductive disorders (Du and Taylor, 2009; Goradel et al., 2018; Liang et al., 2018; Liu et al., 2018; Sneddon et al., 2018); these cells can be harvested from the varieties of tissues including bone marrow, umbilical cord, adipose tissue, placental tissue, menstrual blood and dental pulp (Priester et al., 2020).

In spite of the therapeutic potential of MSCs, large-scale MSC expansion for clinical use is limited owing to the cells’ capacity to divide in culture for a limited number of passages. Also, the cells could be associated with some challenges including difficulty of their transportation, transplant rejection and commercialization (Mendt et al., 2019). Therefore, in the recent decades, great efforts have been taken to find alternatives to reducing problems of MSCs usage while preserving their positive properties.

MSCs are a massive source for exosome production and are used in various research fields due to their greater availability and high proliferative ability (Cheng et al., 2017; Cheng et al., 2021). Exosomes, which are lipid bilayer nanoparticles that secrete into the microenvironment from various types of cells especially mesenchymal stem cells that offer promising therapeutic potential. In addition to other bioactive molecules that we have detailed in our previous study (Izadi et al., 2021), exosomes have various types of signaling molecules such as mRNA and miRNA (Valadi et al., 2007). Higher biological stability, easier storage, easier penetration into target tissues and low immunogenicity are some of the considerable advantages that make exosomes more useful compared to their source cells for medical applications (El Andaloussi et al., 2013; Zhang et al., 2016). Exosomes secreted from different cells have almost similar protein molecules with biological activities including immune modulation, regeneration, and tissue repair and angiogenesis promotion. Exhibiting the same activities in all MSC-derived exosomes may be related to the existence of a common protein signature (van Balkom et al., 2019). Additionally, some types of MSCs secrete exosomes with unique characteristics (Tang et al., 2021). Rising evidence suggests that MSCs-derived exosomes have immunomodulation, anti-inflammatory (Urbanelli et al., 2015; Izadi et al., 2021) and anti-apoptosis effects (Fu et al., 2020; Wen et al., 2020), therapeutic potential of female reproductive disorders (Liao et al., 2021; Zohrabi et al., 2022).

### Potential applications of MSCs-derived exosomes in PCOS patient

Many studies have shown that chronic low-grade, increase in pro-inflammatory cytokines, decrease of anti-inflammatory cytokines, insulin resistance, hypersensitivity of Helper T-cells (Th1); Th1-type immunity and the ratio of Th1 to Th2 cells, as well as Th1 cytokines such as IFN-γ and IL-2 are increased during immune reactions in PCOS patients (Qin et al., 2016) and hyperandrogenism play a crucial roles in PCOS pathogenesis (González et al., 2014a). Moreover, it has been reported that chronic inflammation in PCOS can lead to poor oocyte quality, ovarian dysfunction, disrupts oocyte development, and affect endometrial receptivity (Velez et al., 2021).

Few recent studies have reported that human umbilical cord mesenchymal stem cells (huMSCs) therapy can improve ovarian dysfunction by the systemic immunomodulation and local immune response in the ovary of PCOS patients (Xie et al., 2019). In a letrozole-induced PCOS mouse model, the beneficial effect of human bone marrow derived mesenchymal stem cells (BM-hMSCs)on the partial restoration of ovaries, the number of corpora lutea, and antral follicles has been reported (Chugh et al., 2021a). Notably, an increasing number of studies have discovered that the communication between MSCs and target tissue such as ovarian microenvironment is through the exosomes and secretome (Harrell et al., 2019; Xu et al., 2019). Also, another study reported that huMSCs--derived exosomes ameliorates the granulosa cells immune response through the inhibition of NF-κB signaling pathway in the PCOS (Zhao et al., 2022).

Recently, a study showed that MSCs-derived exosomes cause the decreased concentration of IL-1β and TNF-α, while the secretion of TGF-β increased in *in vitro* culture of mononuclear cells. Also, it demonstrated that MSCs-derived exosomes can increase Th2 (Th2-related anti-inflammatory cytokine such as IL-10 that is reduced in PCOS patients) and Treg and decrease Th1 (Chen et al., 2016). Apoptosis plays the key role in follicular atresia and cyclic growth and regression of follicles in the human ovary (Tilly, 1996). It has been reported that factors involved in the induction of apoptosis in the ovaries (Jansen et al., 2004) and also the number of atretic follicles increase in PCOS patients (Laven et al., 2001). EVs derived from huMSCs have also shown anti-apoptotic and fertility recovery effects and promoted secretive functions of granulosa cells in induced POI mice (Liu et al., 2020). Also it can reduce ovarian damage and protect GCs through anti-apoptotic and anti-inflammatory effects and improve ovarian function in chemotherapy-induced POF mice (Deng et al., 2021).

Therefore, exosome as a novel cell-free therapeutic strategy can be used promisingly in diseases of inflammatory origin by maintaining the immune balance (Chen et al., 2016).

### The effects of bioactive compounds in the MSC-derived exosomes and secretome

Although recently there have been many studies on exosomes as a novel avenue for female infertility treatment, precise mechanisms of MSCs-derived exosomes on female reproductive diseases are also unclear. Given that chronic inflammation is associated with the pathogenesis of PCOS, there is also a positive feedback loop between inflammation, androgen production and metabolic disorders in PCOS (González et al., 2014b; Fox et al., 2019); Since, near to 50% of PCOS patients show high secretion of androgens (Marti et al., 2017; McAllister et al., 2019), therefore, the main strategy to treat PCOS can be suppression of androgen secretion (McAllister et al., 2019).

It has been reported that cytokine IL-10 that is found in secretome improves fertility through the suppressing androgen secretion by ovarian theca cells and reducing inflammation (Chugh et al., 2021b). Bone morphogenetic proteins (BMPs) are multifunctional growth factors that play an important role in folliculogenesis and female fertility; these proteins are secreted by BM-hMSCs (Yoshino et al., 2011). The theca cells in the ovary proliferate rapidly and increased androgen production in PCOS (Bremer, 2010; Zhang et al., 2012), it has been reported that BMP-2 can inhibit the proliferation of different cells *in vitro* (Hardwick et al., 2004; Chen et al., 2012; Zhang et al., 2012). Another study showed that BMP-2 can treat hyperandrogenemia in PCOS by suppressing steroidogenesis (Chugh et al., 2021a). Therefore, BMP-2 may improve the hyper-androgenemia in PCOS.

In PCOS and other ovarian disorders, the effect of exosome therapy has been reported to affect apoptosis by delivering genetic material such as miR-323-3p miR-146a and miR-10a (Xiao et al., 2016; Zhao et al., 2019), miR-664-5p (Sun et al., 2019), and miR-21(90).

**FIGURE 1:**
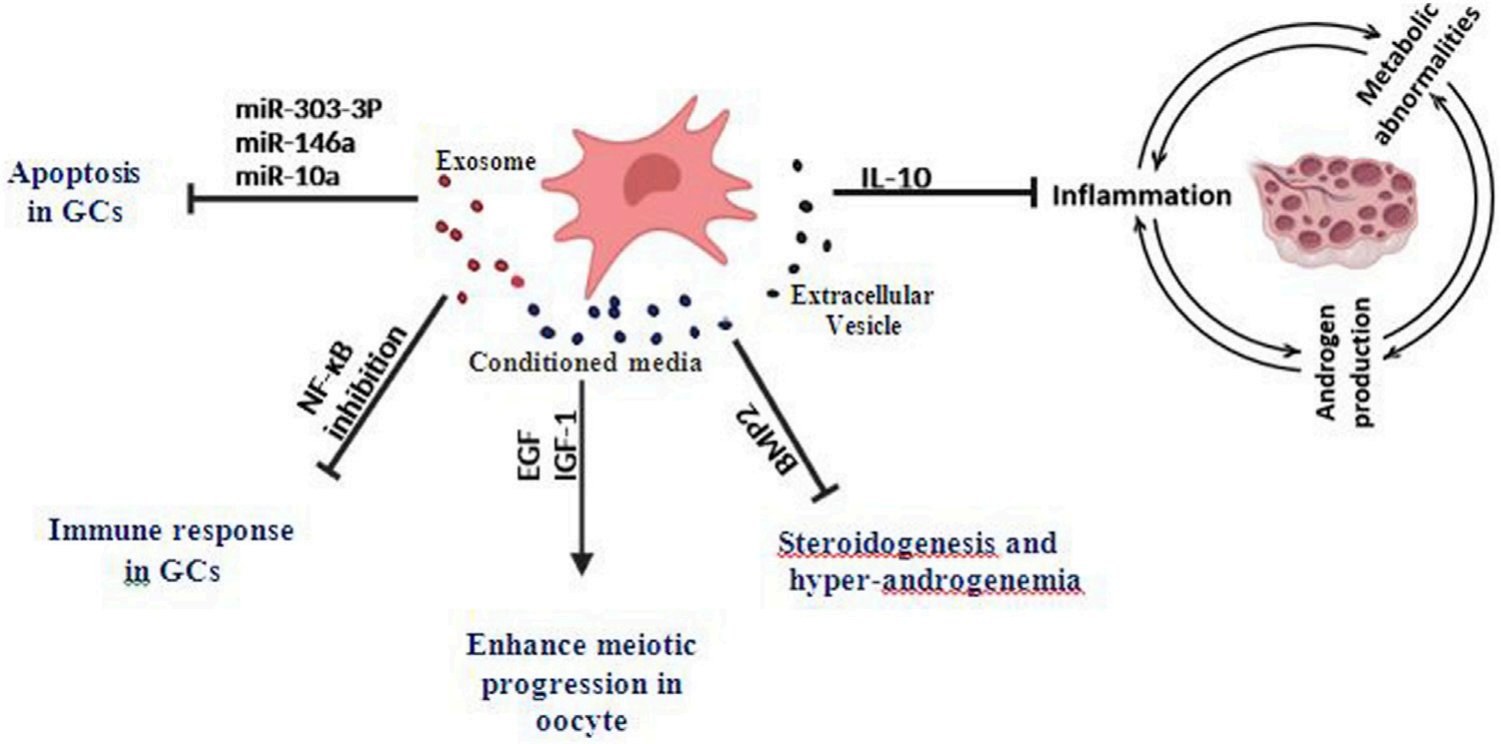
Effects of MSCs-derived exosomes, EVs, and Conditioned media on inflammation and granulosa cells apoptosis in PCOS and various ovarian disorders.

Mesenchymal stem cells have the ability to secrete a large number of growth factors such as fibroblast growth factor (FGF), insulin-like growth factor-1 (IGF-1), VEGF, TGF-β, and EGF (Labouyrie et al., 1999; Izumida et al., 2005; Yoon et al., 2010) which may have the effect of reinitiate meiosis and improve oocyte maturation (Ling et al., 2008). A clinical trial in which the retrograde injection method was used to transplant MSCs based on a collagen scaffold into the ovaries of patients with some ovarian disorders suggests that EVs can be transferred by intra-ovarian injection (Ding et al., 2018). The biologically active molecules and their effects are summarized in Table 1 and Figure 1.

**TABLE 1:** Therapeutic potential of MSC-derived secretome in ovarian and *in vitro* culture.

Secretome type	Bioactive compounds	Source cell type	Target cells or tissue	Effects	Ref. No.
Exosome	miR-323-3p	AMSCs	cumulus cells	Inhibit apoptosis in GCs, regulation of steroidogenesis	Zhao et al. (2019)
	miR-146a, miR-10a	AFSCs	GCs	Inhibit apoptosis in GCs	Xiao et al. (2016)
	miR-664-5p	BMSC	GCs	Inhibit apoptosis in GCs	Sun et al. (2019)
Conditioned media	IL-10	BM-hMSCs	intra-ovarian injection	Reduce inflammation and androgen secretion	Chugh et al. (2021b)
	BMP-2	BM-hMSCs	H295R cells*	Reduce Steroidogenesis	Chugh et al. (2021a)
	EGF, IGF-1	MSCs	Oocyte	Improve oocyte maturation	Ling et al. (2008)
Extracellular vesicle	miR-21	AFMSCs	GCs	Inhibit apoptosis in GCs	Thabet et al. (2020)

BMP, Bone morphogenetic proteins; GCs, granulosa, cells; BM-hMSCs, Human bone marrow mesenchymal stem cells; BMSC, Bone mesenchymal stem cell; AMSCs, Adipose mesenchymal stem cells; AFSCs, Amniotic fluid stem cells; AFMSCs, Amniotic fluid mesenchymal stem cells.

*In vitro cell culture model for androgen production.

### Potential applications of MSCs-derived exosomes in enrichment IVM culture medium

Although *in vitro* fertilization (IVF) is an effective treatment for infertility in PCOS women, it is also associated with an increased risk of ovarian hyperstimulation syndrome (OHSS) (Shalom-Paz et al., 2012). Therefore, minimizing the risk of ovarian stimulation while providing an acceptable fertility success rate should be the focus of treatment efforts. Currently, to prevent OHSS, immature oocytes are collected from small antral follicles within unstimulated or very little stimulated ovaries then these oocytes are matured *in vitro*. Patients with PCOS could potentially benefit from IVM, as it reduces the risk of OHSS as well as costs (Shalom-Paz et al., 2012; Ho et al., 2019). But since the IVM and success rate of fertilization of oocytes matured *in vitro* is not satisfactory, therefore, to overcome these limitations faced by IVM, several studies have been conducted that focus on effects of cultural media containing various additives for improving oocyte quality (Jee et al., 2008; Ben-Ami et al., 2011; Blanco et al., 2011; Ishizuka et al., 2013; Sánchez et al., 2015). Primarily, an optimal culture medium is needed to increase the efficiency of IVM, which can be achieved by better understanding the molecular events that trigger oocyte maturation (Chian et al., 2004). Before ovulation, the LH surge triggers a cascade of cellular and molecular events in the ovarian follicle including resumption of oocyte meiosis, cumulus expansion, follicular wall rupture, and cumulus-oocyte mass extrusion (Richards et al., 2002). Despite mural granulosa cells and external theca cells expressing high LH receptors, oocyte and cumulus cells express little or no LH receptors and therefore do not respond to LH exposure *in vitro* (PENG et al., 1991). Therefore, it seems the effects of LH on cumulus-oocytes may be through the release of the paracrine mediators from granulosa cells (Conti et al., 2006). Also, recently, it was reported that LH stimulation of isolated human granulosa cells causes the increase of EGF-like growth factors (Ben-Ami et al., 2006; Ben-Ami et al., 2009). Several experimental studies in animals and cell culture have demonstrated that EGF and IGF-1 can improve maturation in cumulus surrounded (Sakaguchi et al., 2000; Sakaguchi et al., 2002) and denuded oocytes as well as *in vitro* which is similar to what happens *in vivo* (Das et al., 1991; Lonergan et al., 1996).

Given that each oocyte is surrounded by cumulus granulosa, mural granulosa, theca cells and follicular fluid to form ovarian follicles as reproductive units, therefore, the oocyte can be affected by each of these components (Di Pietro, 2016). However, new exosome-based therapeutic approaches in PCOS are few. Recently, regulation of steroidogenesis, promotion of cell growth and inhibition of apoptosis in the cumulus cells by exosomal miR-323-3p has been reported in the women with PCOS (Zhao et al., 2019). Also, animal studies revealed beneficial effects of EVs (Liao et al., 2021), exosomes derived from amniotic fluid stem cells (Xiao et al., 2016) and bone mesenchymal stem cells (Sun et al., 2019) on various ovarian disorders and fertility recovery. These nanoparticles inhibit apoptosis in the damaged granulosa cells through the delivery of miR-146a, miR-10a (Xiao et al., 2016), miR-664-5p (Sun et al., 2019), and miR-21(90). Studies have shown that exosomes derived from huMSCs can increased of Bcl-2 and caspase-3 whereas decreased the expression of Bax, cleaved caspase-3, and cleaved poly (ADP-ribose) polymerase (PARP) to attenuation of cisplatin-induced ovarian granulosa cell apoptosis *in vitro* (Sun et al., 2017; Zhang et al., 2020). In addition, BM-hMSCs conditioned media could regulate the steroidogenesis, inhibit androgen secretion and suppress inflammatory pathways in a cellular model (Chugh et al., 2021a; Chugh et al., 2021b). Moreover, it has been reported that *in vitro* maturation of mouse oocytes with or without cumulus cells can be improved by its co-culture with conditioned medium of MSCs (Ling et al., 2008). Therefore, According to the beneficial effects of MSCs-derived exosomes, EVs and secretome can have the potential to optimize the culture media for oocyte maturation in PCOS.

## Conclusion

Although the pathogenesis of PCOS is still controversial and remains unclear, several studies implicate chronic inflammation in the pathogenesis of PCOS and others implicate irregular granulosa cell apoptosis in PCOS infertility. In this study, we present a promising opportunity to develop novel cell-free therapy approaches to restore fertility in PCOS condition. According to the recent studies, MSCs-derived exosomes, EVs and secretomes inhibit inflammation and apoptosis, regulate steroidogenesis and inhibit androgen production in *in vitro* as well as *in vivo*. Consequently, it is worthwhile to challenge the effectiveness and efficiency of the exosomes in enriched culture media for improving oocyte development as well as PCOS treatment.
